# Structural Changes in Brain Magnetic Resonance Imaging Associated With Psychogenic Non-epileptic Seizures: An Analytical Cross-Sectional Study

**DOI:** 10.7759/cureus.32144

**Published:** 2022-12-02

**Authors:** Imran Khawaja

**Affiliations:** 1 Department of Internal Medicine, Ayub Teaching Hospital, Abbottabad, PAK

**Keywords:** seizure, psychogenic functional non-epileptic seizures, epilepsy, pnes, magnetic resonance imaging

## Abstract

Background

Psychogenic non-epileptic seizures (PNES) are often seen as indicators of poor motor and sensory function caused by psychological responses to stressful experiences. A seizure might trigger these reactions. The aim of our study was to assess the structural changes in brain MRI associated with psychogenic non-epileptic seizures.

Methodology

A retrospective analytical cross-sectional study at the Department of Medicine and Neurology, Ayub Teaching Hospital, Abbottabad, was conducted from October 2020 to September 2021. The medical records of patients with confirmed PNES were collected and retrospectively evaluated.

Results

Medical records and MRI scans were accessible for 52 patients with PNES; 10 patients were excluded from the study. The average age of the patients (standard deviation) was 34 (±9) years, and the average age at onset was 31.6 (±5.8) years. Based on the video-EEG recordings, 57.1% of patients (n=24) were classified as having broadly generalized motor seizures, 40% of patients (n=17) were classified as having predominantly akinetic seizures defined primarily by blank spells, and only one patient was classified as having focal motor seizures. Only three patients (7%) had a positive epilepsy family history. Twenty-four (47.6%) patients with brain MRI scans reported abnormal findings, while 22 (52.4%) had normal MRI findings. The majority of patients with abnormal MRIs had nonspecific white matter changes (50%), mesial temporal sclerosis (15%), and cysts (15%). In a statistical analysis, age at the beginning of PNES (p = 0.04), duration of PNES (p=0.01), concomitant epilepsy (p = 0.05), generalized motor seizures (p= 0.03), and focal motor seizures (p= 0.02) were strongly associated with abnormal brain MRI findings.

Conclusion

Research reveals that persons with PNES have a higher-than-average prevalence of anatomical brain abnormalities. The main takeaway is that these findings lend credence to the growing body of data suggesting that PNES may not be a medical mystery but rather a disorder with physical foundations in the brain. Important implications for diagnosing and treating PNES patients are discussed, as are the outcomes of earlier neuroimaging investigations of PNES. Studying the involvement of structural brain anomalies in the etiology of psychogenic non-epileptic seizures requires further well-designed multicenter studies with larger sample sizes and a consistent imaging approach (PNES). It is crucial to consider any confounding variables, such as co-occurring mental diseases, while designing this study.

## Introduction

Uncoordinated electrical impulses between neurons cause epileptic seizures; however, not all seizures result from abnormal neuronal activity. Mental symptoms and psychosocial pressures produce psychogenic non-epileptic (functional) seizures (PNES). Electroencephalograms (EEGs) remain normal despite the clinical resemblance of paroxysmal bouts of PNES to epileptic fits. Also, specific anticonvulsant drugs (ACMs) do not treat PNES and might even worsen it. Due to the same semiology and failure of anticonvulsant drugs, the first diagnosis for many individuals with PNES is treatment-resilient epilepsy (TRE) [1]. PNES patients, like those with TRE, have increased rates of anxiety, depression, past abuse, trauma, and poor quality of life [2-4]. Research has examined mental comorbidities and psychosocial stresses in PNES subjects [5,6]. However, less research is available about the neurological correlates of PNES. Although they are seen etiologically as psychological manifestations, the neuro-pathophysiology of PNES is an important research topic [7].

According to neuroimaging research, PNES is a condition of many extensive neural networks categorized by multifocal brain activity and connection impairments [8,9]. Consecutively, these are associated with their morphology and cytoarchitecture. Limited research has examined the morphological implications of PNES to date. The absence of gross anomalies on magnetic resonance imaging (MRI) has led many to the conclusion that the brain morphology of PNES patients is expected, which is correct from a "lesional standpoint" [10]. However, the absence of macroscopic structural MRI abnormalities in PNES implies that network problems may result from microstructural disease, which may be studied using the latest computational approaches like voxel-based morphology (VBM) and surface-based morphology (SBM) [11-13]. So far, only two relatively modest investigations have employed different computational approaches to examine the brain morphology of PNES patients. The initial examination of brain anatomy in PNES (n = 20) revealed cortical atrophy of the suitable motor and premotor areas and the bilateral cerebellum, compared to 40 healthy controls with similar characteristics. Higher depression levels were connected with premotor area atrophy [1]. The left inferior anterior insula, bilateral orbitofrontal, and precentral cortical thickness increased. The second study found that it dropped in the left anterior cingulate, which included 37 PNES patients and 37 matched controls for age and gender [14,15].

The inconsistency of previous study results emphasizes the need for more studies describing PNES-specific structural modifications. Neuroplasticity direction (growth vs. damage) is often conserved across patient groups. Based on the clinical implications, prior research on PNES, and analyses in persons with psychological disorders, we would expect neuronal loss and cortex shrinkage in brain areas involved in emotional self-regulation. Previous research has not shown structural imaging abnormalities [16]. This study also advanced prior research on brain imaging abnormalities in PNES patients. In these individuals, structural brain MRI anomalies may be associated with several clinical variables, most notably seizure severity. We expected that morphological abnormalities associated with psychological problems would result in volume- or surface-based neuronal loss corresponding to patients’ self-reported impact and functioning. The present research aims to study the structural changes in the brain associated with psychogenic non-epileptic seizures.

## Materials and methods

A retrospective analytical cross-sectional study at the Department of Medicine and Neurology, Ayub Teaching Hospital, Abbottabad, was conducted from October 2020 to September 2021. The purposive sampling technique was used to select patients with PNES from the department database.

We looked back at the clinical data of people with PNES who were being treated and evaluated at the Neurology and Medicine department of the Ayub Teaching Hospital in Abbottabad. The diagnosis of PNES was made when the thorough clinical history fit with the diagnosis; the neurologist saw seizures that looked like PNES caused them. At the same time, they were being recorded on video electroencephalograph (video-EEG), and there was no interictal paroxysmal activity right before, during, or after the attack on the video-EEG recording. A detailed clinical history was also retrieved to see if epileptic seizures were happening simultaneously. The EEG that was taken between seizures was carefully looked over to look for any possible epileptiform expulsions. We do not usually perform magnetic resonance imaging (MRI) of the brain on patients with PNES. However, many of these people have already had an MRI of the brain before being sent to our department. The radiologists who perform the brain MR tests are unaware of the definitive epilepsy classification vs. psychogenic non-epileptic seizures; nonetheless, they know that the patient suffers seizures.

Participants of both genders and any age were included in the study. Participants in the study were required to meet the following criteria: (1) PNES is confirmed on video-EEG monitoring; (2) video-EEG monitoring does not show epileptic seizures or epileptic discharges. Patients with a significant history of acute encephalopathy, meningitis, severe head injury, or hypoxic encephalopathy, as well as a low Wechsler Adult Intelligence Scale III intelligence quotient, were excluded from the research.

Forty-two MRI brain scans of patients with confirmed PNES were retrieved and reviewed retrospectively, and participants were based purely on our study’s inclusion/exclusion criteria. Individuals with available 3T T1-weighted brain MRIs were analyzed to discover if specific clinical variables are connected with anatomical brain imaging anomalies in these patients. Patient age, age at seizure onset, gender, seizure characteristics (frequency and semiology), factors potentially predisposing to PNES (history of physical, emotional, and verbal abuse), history of head injury, and a family history of epilepsy and comorbid epilepsy and psychiatric complications were collected from the medical records of patients.

Statistical Package for the Social Sciences (SPSS) V-26 was used for data analysis (IBM Corp., Armonk, NY). The significance level (alpha) cut-off was set at <0.05. If the variables were non-discrete or quantitative, all data were given as mean and standard deviation. Frequency and proportions were used to represent discrete and categorical data. Fisher's exact and Mann-Whitney U tests were used to assess the relationships between brain MRI and the demographic and clinical factors of the sample.

## Results

In the present study, our database had the medical records of 130 patients, of whom 52 (40%) received a brain MRI and 78 (60%) did not. Only 42 patients who met our study’s requirements were selected. The patients’ average (SD) age was 34 (± 9) years, and the mean age at PNES onset was 31.6 (±5.8). 57.1% of patients (n=24) were classified as having largely generalized motor seizures, 40% of patients had mainly akinetic seizures (n=17), and only one patient was classified as having focal motor seizures based on the video-EEG recordings. Only three patients (7%) had a positive epilepsy family history. Table 1 shows the demographics and clinical features of the patients.

**Table 1:** The demographics and clinical features of the patients. N=42, PNES: psychogenic non-epileptic seizures, GMS: generalized motor seizures, OCD: obsessive-compulsive disorder.

Variable	n (%)	Mean ±SD
Age		34 ± 9
Gender		
Female	24 (57.1%)	
Male	18 (42.9%)	
Family history of epilepsy		
Negative	39 (92.9%)	
Positive	3 (7.1%)	
Age at onset of PNES		31.6±5.8
Duration of PNES		4.6 ± 6.3
Frequency of seizures		33.1±14.8
PNES severity symptoms score		2.6±1.6
Comorbid epilepsy	25 (59.5%)	
GMS	24 (57.1%)	
Focal motor seizures	1 (2.4%)	
Akinetic seizures	17 (40.5%)	
History of abuse		
Physical	30 (32.6%)	
Verbal	30 (32.6%)	
Emotional	32 (34.8%)	
Psychiatric complications		
Depressive disorders	22 (40.7%)	
Anxiety disorders	20 (37.0%)	
OCD	12 (22.2%)	

A competent neuroradiologist visually examined the brain MRI of each patient for indications of brain injury or pathological brain abnormalities. Twenty patients (47.6%) reported aberrant findings in their brain MRI scans, and twenty-two patients (52.4%) had normal imaging studies. Table 2 displays the results of brain MRIs performed on these patients. The majority of patients with an abnormal MRI had non-specific white matter lesions (50%), hippocampal sclerosis (15%), and suprasellar arachnoid cysts (15%).

**Table 2:** MRI brain scans findings in patients with psychogenic non-epileptic seizures.

MRI finding	n	%
Normal	22	52.2%
Abnormal	20	47.6%
White matter non-specific lesions	10	50.0%
Brain tumor	0	0
Hippocampal sclerosis	3	15.0%
Astrocytosis and encephalomalacia	0	0
Suprasellar arachnoid cyst	3	15.0%
Parenchymal atrophy	2	10.0%
Congenital anomalies	0	0
Vascular lesion	1	5.0%
Demyelinating lesion	1	5.0%

We examined the relationships between brain MRI and demographic and clinical factors using Fisher’s exact test (Table 3). A statistical test revealed that age at the beginning of PNES (p<0.05), duration of PNES (p<0.05), generalized motor seizures (p<0.05), and focal motor seizures (p<0.05) were substantially linked with aberrant brain MRI results.

**Table 3:** Associations between demographic and clinical characteristics and MRI findings. N=42, PNES: psychogenic non-epileptic seizures, GMS: generalised motor seizures, OCD: obsessive compulsive disorder. *Significant result.

Parameters	MRI normal (n=22)	MRI abnormal (n=20)	P-value
Mean age	34.8±9.7	33.3±8.5	0.92
Age at PNES onset	32.1±5.7	31.2±6.2	0.04*
Duration of PNES	3.5 ±7.2	5.1 ± 9.2	0.01*
Frequency of seizures	35.4±17.5	30.7±11.1	0.851
PNES severity symptoms score	2.7±1.6	2.6±1.7	0.427
Family history	0	3 (15%)	0.099
Comorbid epilepsy	13 (59%)	12 (60%)	0.05*
GMS	14 (64%)	10 (50%)	0.02*
Akinetic seizures	8 (36%)	9 (45%)	0.8
Focal motor seizures	0 (0)	1 (5%)	0.03*
H/O physical abuse	17 (77%)	13 (65%)	0.152
H/O verbal abuse	17 (77%)	13 (65%)	0.49
H/O emotional abuse	19 (86%)	13 (65%)	0.15
Depressive disorders	10 (46%)	12 (60%)	0.374
Anxiety disorders	10 (45%)	10 (50%)	0.15
OCD	7 (32%)	5 (25%)	0.738

## Discussion

Only about a quarter of the 130 participants with PNES in this study had a brain MRI. In most cases, it is clinically reasonable and appropriate to do a magnetic resonance imaging scan of the brain of individuals with epilepsy [17]. Sixty percent of patients in recent studies of 224 people with PNES solely had brain MRI [18]. The authors found that the profile of individuals experiencing psychogenic non-epileptic seizures was correlated with the number of times they underwent neurodiagnostic testing. Our results differed from those of earlier research. Their different methods of operation might explain the apparent difference between these research projects.

Twenty patients (47.6%) out of 42 with PNES in this study had abnormal brain MRIs. Most prior investigations [16,18-21] corroborate our results. Twenty-two percent of those with PNES in a 10-year U.S. cohort study had abnormalities on their brain MRI scans [21].

In a study from the United Kingdom, biomarkers of brain abnormalities in people with PNES were analyzed to see whether or not they were associated with an increased risk of PNES. Electrical and imaging abnormalities in the brain were more prevalent in the PNES + E group (epileptiform potentials in 70.7%, MRI changes in 60.2%, and NPS deficits in 52.8%; p=0.0001) [19]. These results support the hypothesis that structural abnormalities in the brain contribute to the development of the illness [20,22]. One hundred and thirty-two individuals with PNES were assessed between 2008 and 2019 at the Shiraz Comprehensive Epilepsy Center in Iran, and their medical records were reviewed for this research. They found that 36% of individuals had abnormalities visible on MRI. In addition to age, having a history of epilepsy was also significantly associated with MRI abnormalities of the brain. Out of the remaining 86 people (who just had PNES), only 23 got normal MRI findings. Combination PNES/epilepsy patients were more likely to have epileptogenic structural brain abnormalities (such as tumors, mesial temporal sclerosis, encephalomalacia, and developmental anomalies [23]. However, previous studies, including ours, lacked adequacy due to being ex post facto and not including healthy controls [16-20].

Our findings showed that about half of PNES patients exhibited epileptogenic brain abnormalities such as white matter nonspecific lesions, hippocampal sclerosis, suprasellar arachnoid cysts, and parenchymal atrophy. Similar results have been found in the past [18-20]. These researchers evaluated the results of MRI scans taken from those who only had PNES and those who experienced both PNES and epilepsy. In contrast, 47.6% of PNES patients had abnormal brain MRIs, while 52.4% had normal MRIs. Previous research demonstrates that variables other than physical brain defects substantially influence the onset and maintenance of PNES [23-25]. Our result findings show that abnormal brain MRI findings in individuals with PNES were strongly correlated with age at the start (p=0.01), duration (p=0.01), concomitant epilepsy (p = 0.05), generalized motor seizures (p=0.03), and focal motor seizures (p=0.02). These findings make complete sense and are consistent with prior research and hypotheses [16]. Individuals with coexisting epilepsy were more likely to exhibit aberrant brain abnormalities. In addition, it is hypothesized that individuals in their latter years will have a higher rate of brain imaging abnormalities (e.g., atrophy, cysts, and nonspecific white matter changes, frequently observed in our study). In our research, we found no evidence that any clinical patient characteristics were linked to the presence of brain MRI abnormalities. Unfortunately, we could not examine lateralization or any particular morphological anomalies in our investigation owing to the non-availability of the latest computational approaches like VBM and SBM [11-13]. Neuroimaging has shown that patients with significant depressive disorders have structural disruptions in the white and grey matter of the brain [23-26]. Lesions in the non-dominant hemisphere, as opposed to the dominant hemisphere, have been linked to an increased risk of PNES [26], while other researchers have contested this finding [20]. "The absence of clear hemispheric dominance or lobar predominance from this research supports the concept of a heterogeneous etiology and phenomenology of PNES," concluded a recent thorough and systematic assessment of brain imaging studies in people with PNES. The high frequency of mental comorbidities in people with PNES lends credibility to the hypothesis that the underlying brain abnormalities in PNES are distinct compared to those in patients with other psychopathologies [20]. The findings of our study have to be seen in light of some limitations. First is the relatively small sample size, so findings cannot be generalized to a broader population, and there is a need to conduct studies with larger sample sizes. The second limitation is the retrospective study design; therefore, the findings are subject to biases and confounding. Third is that in our study, we could only evaluate MRI findings alone owing to the non-availability of the latest computational approaches like VBM and SBM. More longitudinal morphometric investigations are required to determine if morphological alterations in the brain are a cause or effect of PNES. Furthermore, PNES are transient occurrences, making them challenging to study using MRI data alone. EEG ictal data recorded during non-epileptic episodes should be mapped to the underlying structure, connectivity, and folding patterns of the cerebral cortex to complete the picture beyond what is provided by interictal data. Potentially, this will help us better understand the pathophysiological processes underlying PNES. It is too soon to draw a definitive conclusion; nonetheless, the finding of the frequent occurrence of structural brain abnormalities in individuals suffering PNES underlines the need for future investigation to test this logical hypothesis acceptably.

## Conclusions

Research reveals that persons with PNES have a higher-than-average prevalence of anatomical brain abnormalities. The main takeaway is that these findings lend credence to the growing body of data suggesting that PNES may not be a medical mystery but rather a disorder with physical foundations in the brain. Important implications for diagnosing and treating PNES patients are discussed, as are the outcomes of earlier neuroimaging investigations of PNES. Studying the involvement of structural brain anomalies in the etiology of psychogenic non-epileptic seizures requires further well-designed multicenter studies with larger sample sizes and a consistent imaging approach. It is crucial to consider any confounding variables, such as co-occurring mental diseases, while designing this study.
